# Research priorities to reduce the impact of COVID-19 in low- and middle-income countries

**DOI:** 10.7189/jogh.12.09003

**Published:** 2022-04-15

**Authors:** Ozren Polašek, Kerri Wazny, Davies Adeloye, Peige Song, Kit Y Chan, Danladi A Bojude, Sajjad Ali, Sheri Bastien, Francisco Becerra-Posada, Florencia Borrescio-Higa, Sohaila Cheema, Darien A Cipta, Smiljana Cvjetković, Lina D Castro, Bassey Ebenso, Omolade Femi-Ajao, Balasankar Ganesan, Anton Glasnović, Longtao He, Jean M Heraud, Chinonso Igwesi-Chidobe, Per O Iversen, Bismeen Jadoon, Abdulkarim J Karim, Johra Khan, Raaj K Biswas, Giuseppe Lanza, Shaun WH Lee, You Li, Li-Lin Liang, Mat Lowe, Mohammad M Islam, Ana Marušić, Suleiman Mshelia, Anthony M Manyara, Mila NN Htay, Michelle Parisi, Prince Peprah, Emma Sacks, Kabiru O Akinyemi, Fariba Shahraki-Sanavi, Konstantin Sharov, Elena S Rotarou, Srdjan Stankov, Wenang Supriyatiningsih, Benjamin TY Chan, Mark Tremblay, Dialechti Tsimpida, Sandro Vento, Josipa V Glasnović, Liang Wang, Xin Wang, Zhi X Ng, Jianrong Zhang, Yanfeng Zhang, Harry Campbell, Mickey Chopra, Simon Cousens, Goran Krstić, Calum Macdonald, Parisa Mansoori, Smruti Patel, Aziz Sheikh, Mark Tomlinson, Alexander C Tsai, Sachiyo Yoshida, Igor Rudan

**Affiliations:** 1 Department of Public Health, University of Split School of Medicine, Split, Croatia; 2 Croatian Centre for Global Health, University of Split, Croatia; 3 Algebra University College, Zagreb, Croatia; 4 Children's Investment Fund Foundation, London, UK; 5 Centre for Global Health, Usher Institute, The University of Edinburgh, UK; 6 School of Public Health and Women’s Hospital, Zhejiang University School of Medicine, China; 7 Gombe State University, Gombe, Nigeria; 8 Department of Medicine, Ziauddin Medical University, Karachi, Pakistan; 9 Norwegian University of Life Sciences, Ås, Norway; 10 Public Health Development Organization, El Paso, Texas, USA; 11 Universidad Adolfo Ibañez, Santiago, Chile; 12 Weill Cornell Medicine – Qatar, Doha, Qatar; 13 Universitas Pelita Harapan, Jakarta, Indonesia; 14 Faculty of Medicine, University of Belgrade, Serbia; 15 National Institute of Psychiatry Ramón de la Fuente Muñiz, Mexico City, Mexico; 16 Leeds Institute of Health Sciences, University of Leeds, UK; 17 Faculty of Biology, Medicine and Health, University of Manchester, UK; 18 Department of Rehabilitation Sciences, The Hong Kong Polytechnic University, Hong Kong; 19 Croatian Institute for Brain Research, Zagreb University School of Medicine, Zagreb, Croatia; 20 Southwestern University of Finance and Economics, Chengdu, China; 21 Institut Pasteur de Dakar, Dakar, Senegal; 22 University of Nigeria, Enugu Campus, Nigeria; 23 Department of Nutrition, University of Oslo, Norway; 24 Egyptian Representative, Committee of Fellows of Obstetrics and Gynaecology, Oxford, UK, Royal Berkshire Hospital, NHS, UK; 25 College of Veterinary Medicine, University of Baghdad, Iraq; 26 Department of Medical Laboratory Sciences, College of Applied Medical Sciences, Majmaah University, Al Majmaah, Saudi Arabia; 27 University of New South Wales, Sydney, Australia; 28 Oasi Research Institute-IRCCS, Troina, Italy; 29 University of Catania, Catania, Italy; 30 Monash University Malaysia, Selangor, Malaysia; 31 School of Public Health, Nanjing Medical University, China; 32 National Yang Ming Chiao Tung University, Taipei, Taiwan; 33 Society for the Study of Women's Health, Kanifing, The Gambia; 34 University of Dhaka, Dhaka, Bangladesh; 35 Department of Research in Biomedicine and Health, University of Split School of Medicine, Split, Croatia; 36 Jos University Teaching Hospital, Nigeria; 37 University of Glasgow, Glasgow, UK; 38 Department of Community Medicine, Faculty of Medicine, Manipal University College Malaysia, Melaka, Malaysia; 39 Clemson University, South Carolina, USA; 40 Social Policy Research Centre/Centre for Primary Health Care and Equity, University of New South Wales, Sydney, Australia; 41 Johns Hopkins Bloomberg School of Public Health, Baltimore, USA; 42 Lagos State University, Ojo, Lagos, Nigeria; 43 Health Promotion Research Center, Zahedan University of Medical Sciences, Iran; 44 Koltzov Institute of Developmental Biology of Russian Academy of Sciences, Moscow, Russia; 45 Universidad San Sebastián, Santiago, Chile; 46 Pasteur Institute Novi Sad, Novi Sad, Serbia; 47 Muhammadiyah University of Yogyakarta, Yogyakarta, Indonesia; 48 Hong Kong Metropolitan University, Hong Kong; 49 CHEO Research Institute, Ottawa, Canada; 50 University of Puthisastra, Phnom Penh, Cambodia; 51 Department of Hematology, Dubrava University Hospital, Zagreb, Croatia; 52 Xuzhou Medical University, Yuzhou, China; 53 School of Biosciences, Faculty of Science and Engineering, University of Nottingham Malaysia, Semenyih, Malaysia; 54 University of Melbourne, Melbourne, Australia; 55 Capital Institute of Pediatrics, Beijing, China; 56 The World Bank, Washington, District of Columbia, USA; 57 London School of Hygiene and Tropical Medicine, London, UK; 58 International Society of Global Health, Edinburgh, UK; 59 National Institute for Health Research, London, UK; 60 Editor, Journal of Global Health Reports, Washington, DC, USA; 61 Usher Institute, University of Edinburgh, Edinburgh, UK; 62 Stellenbosch University, Cape Town, South Africa; 63 Massachusetts General Hospital, Boston, Massachusetts, USA; 64 World Health Organization, Geneva, Switzerland

## Abstract

**Background:**

The COVID-19 pandemic has caused disruptions to the functioning of societies and their health systems. Prior to the pandemic, health systems in low- and middle-income countries (LMIC) were particularly stretched and vulnerable. The International Society of Global Health (ISoGH) sought to systematically identify priorities for health research that would have the potential to reduce the impact of the COVID-19 pandemic in LMICs.

**Methods:**

The Child Health and Nutrition Research Initiative (CHNRI) method was used to identify COVID-19-related research priorities. All ISoGH members were invited to participate. Seventy-nine experts in clinical, translational, and population research contributed 192 research questions for consideration. Fifty-two experts then scored those questions based on five pre-defined criteria that were selected for this exercise: 1) feasibility and answerability; 2) potential for burden reduction; 3) potential for a paradigm shift; 4) potential for translation and implementation; and 5) impact on equity.

**Results:**

Among the top 10 research priorities, research questions related to vaccination were prominent: health care system access barriers to equitable uptake of COVID-19 vaccination (ranked 1st), determinants of vaccine hesitancy (4th), development and evaluation of effective interventions to decrease vaccine hesitancy (5th), and vaccination impacts on vulnerable population/s (6th). Health care delivery questions also ranked highly, including: effective strategies to manage COVID-19 globally and in LMICs (2nd) and integrating health care for COVID-19 with other essential health services in LMICs (3rd). Additionally, the assessment of COVID-19 patients’ needs in rural areas of LMICs was ranked 7th, and studying the leading socioeconomic determinants and consequences of the COVID-19 pandemic in LMICs using multi-faceted approaches was ranked 8th. The remaining questions in the top 10 were: clarifying paediatric case-fatality rates (CFR) in LMICs and identifying effective strategies for community engagement against COVID-19 in different LMIC contexts.

**Interpretation:**

Health policy and systems research to inform COVID-19 vaccine uptake and equitable access to care are urgently needed, especially for rural, vulnerable, and/or marginalised populations. This research should occur in parallel with studies that will identify approaches to minimise vaccine hesitancy and effectively integrate care for COVID-19 with other essential health services in LMICs. ISoGH calls on the funders of health research in LMICs to consider the urgency and priority of this research during the COVID-19 pandemic and support studies that could make a positive difference for the populations of LMICs.

Research priority setting has been an important process in shaping the global health agenda in the 21st century [1]. The coronavirus disease 2019 (COVID-19) pandemic has changed the agenda for the global health research community and shifted priorities to respond to this threat. More than 220 000 papers on the severe acute respiratory syndrome-coronavirus 2 (SARS-CoV-2) and the COVID-19 pandemic have been published on PubMed alone in 2020 and 2021, which is an unprecedented phenomenon in global health research [2]. However, populations in low- and middle-income countries (LMICs) have been disproportionately affected by the pandemic, which brought about an additional burden on their already weak health systems. This burden has been paired with the lack of quality data, lack of funding for research that could assist policy-making, and lack of a structured framework for setting research priorities [3]. With the large majority of the global population living in LMICs, in addition to global inequity in vaccine distribution, we should also expect that there will be inequity in research funding invested to reduce the impact of COVID-19 in less developed parts of the world [4], especially in Africa [5,6].

The share of research funding for studying COVID 19 in LMICs is likely to be modest in comparison to the share available in high-income countries (HICs). Efforts to track funding flows in real-time are welcome, as they may expose the inequitable distribution of funding and encourage funding from international funders and agencies concerned primarily with global health [7,8]. Differences in research funding and capacity are already quite apparent from the two years of academic publishing of research on COVID-19. According to the Web of Science’s Core Collection database, the United States was the place of origin of more than 65 000 papers, while England, China and Italy contributed about 20 000 each. Spain, Germany, France, Canada, and Australia have each contributed between 8000-11 000 papers to date. The leaders among LMICs are India (about 15 000), Brazil (about 5500), Turkey (about 6500), Iran (about 5300), Pakistan (about 3200), and South Africa (about 3000) [9]. These numbers show that the population size of each country plays a role in the research capacity and productivity, but not nearly as much as the country’s economic potential and investments in health research.

After two years of the pandemic, during which intense learning about the new virus and the disease that it causes occurred globally, there are many avenues in which further research efforts could be placed – especially in LMICs, where there are still many unknowns even about basic epidemiology, the number of deaths from COVID-19, and post-COVID-19 disability. Strategies for setting research priorities to study COVID-19 in LMICs have so far used web-based surveys [10,11], expert input [3], the Delphi method [12], or mixed methods [13]. The Child Health and Nutrition Research Initiative (CHNRI) method, which is the most used in the 21st century for research priority setting [1], has been used in relation to COVID-19 in at least four exercises to date: to set global research priorities on COVID-19 for maternal, newborn, child and adolescent health [14], to improve maternity services [15], to increase vaccination coverage in Europe [16], and to address the long-term sequelae of COVID-19 for patients with pre-existing and new-onset airways disease [17]. The aim of this study was to use the CHNRI method among the members of the International Society of Global Health (ISoGH) to identify research priorities for addressing the burden of COVID-19 in LMICs.

## MATERIALS AND METHODS

This study was based on the CHNRI process, which meant first establishing a pool of experts by inviting active ISoGH members to participate in the priority-setting process based on crowdsourcing. The members were invited to join ISoGH as a result of their publication as the lead or senior author in one of the leading international peer-reviewed medical, public, or global health journals over the last 5 years, which implies their expertise in global public health. A summary of the CHNRI method is given in **Box 1**.

Box 1 **The CHNRI method in brief**

The CHNRI method is a systematic, transparent, and democratic approach to priority setting for health research and health interventions [18,19]. It is based on a collective opinion obtained from the participants who are experts in the field. It is based on crowdsourcing, through which many research ideas are proposed and scored against a pre-defined set of priority-setting criteria. The submitted informed opinions from a larger number of experts can expose the strengths, weaknesses, and relative ranking of each proposed research idea to research funders and policymakers [19]. While the CHNRI method allows researchers to independently generate and score research questions, it also has mechanisms to involve relevant stakeholders - including patients, carers and support groups - ensuring their ownership in the outcomes [18,20-22].

The CHNRI method has been used in more than 100 published studies led by multilateral organisations (eg, World Health Organization (WHO), United Nations Children's Fund (UNICEF)), national governments (eg, China, India, and South Africa), and funders (eg, The Bill and Melinda Gates Foundation) to set research priorities in areas ranging from the reduction of global child mortality, chronic non-communicable diseases or disability to the efficient execution of national health plans [23,24]. The recognised advantages of this method include: (i) its systematic nature; (ii) transparency and replicability; (iii) clearly defined context and criteria; (iv) involvement of the funders, stakeholders and policymakers; (v) a structured way of obtaining information; (vi) informative and intuitive quantitative outputs; (vii) studying the level of agreement over each proposed research idea; and (viii) independent scoring of many experts, thus limiting the influence of individuals on the rest of the group [18,20-25]. Previous experiences and statistical simulations found considerable convergence of collective expert opinion leading to stable and replicable results [25].

### The Management and Consultation Group

We established the Management and Consultation Group (MCG) affiliated with the ISoGH Secretariat with the task of identifying research priorities for addressing the burden of the COVID-19 pandemic in LMIC. In September 2021, we developed a protocol to guide this process in line with recently published revised guidelines for the application of the CHNRI method, based on the experience of its use [18,20-26]. The MCG included 17 authors of this report (IR, OP, DA, PS, KW, KYC, HC, MC, SC, GK, CM, PM, SP, AS, MT, ACT, and SY). This group was selected by gathering academics and professionals who were either: 1) involved with the CHNRI method from the start of its development; 2) were adopters and users of the CHNRI method; or 3) were involved in methodological improvements of the CHNRI process. The MCG coordinated the steps of the priority setting exercise and provided important intellectual input to this report.

### Invitation of experts

This was an ISoGH research priority setting exercise, conducted among its active members. ISoGH is a not-for-profit enterprise based in Edinburgh, UK, with an active interest in setting global health research priorities, developing capacity for health research in LMICs, and providing open-access platforms for knowledge dissemination [27]. The MCG invited 642 active members of the ISoGH from across the world, from more than 100 countries. In the first phase of the invitation, e-mails were sent to the members, seeking their participation and providing the details of the objectives and context of the exercise.

In the second e-mail, ISoGH members that showed interest were invited to generate a minimum of three research questions that they considered a research priority for addressing the burden of COVID-19 in LMICs. A total of 79 of 642 ISoGH members responded by providing three or more research questions, leading to more than 200 initial research questions.

The MCG then scrutinised the submitted research questions, removed duplicate questions, and ensured that the wording of each research question allowed its scoring against pre-defined criteria. This led to a consolidated list of 192 unique research questions. Then, the ISoGH members were re-invited to systematically score these ideas using the five pre-agreed priority setting criteria (detailed below).

A total of 52 active ISoGH members provided their scores. Brief job title and/or job description, affiliation to HIC or LMIC countries, and the number of years of experience working in global health are presented for 52 scorers and 17 MCG members in Table S2 in the **Online Supplementary Document**.

One of the main motivations behind the founding of the ISoGH was to gather many experts from LMICs and enable them to have a voice on the research priorities through the CHNRI exercise. This is reflected in 27 of 52 scorers being affiliated to one of the LMICs, which is a majority of scorers.

### Research context and criteria

The context and the criteria for scoring were defined in line with recommendations from the previous exercises and guidelines [18,20-26]. The geographic context was defined as LMICs, taking into account that the focus of interest of the ISoGH is the research capacity and COVID-19 burden in LMICs. The timeframe within which the results were expected from the proposed research was specified as “urgent”, given the urgency of the impact of the COVID-19 pandemic on LMICs. The age group of people affected by COVID-19 was defined as “all ages”, while the target population included “all populations living in LMICs”.

MCG members carefully considered the context of this exercise according to the CHNRI framework and revised the criteria that were most commonly used in the previous CHNRI exercises [18,20-25]. Based on this analysis and further consultations, five independent criteria were agreed upon by the MCG members for this exercise. They were then used to discriminate between the many proposed research questions. Those criteria are:

1. Feasibility and answerability – “Would you say that the proposed research would likely be feasible and successful in reaching the proposed endpoint?”
2. Potential for burden reduction – “Would you say that this research has the potential to markedly reduce the burden of COVID-19 pandemic on patients, caregivers, and society in LMICs?”
3. Potential for a paradigm shift – “Would you say that this research is likely to result in a “paradigm shift” that could change and improve our current understanding of the problem of COVID-19 in LMICs?”
4. Potential for translation and implementation – “To the best of your knowledge and experience, would you say that the proposed research would likely lead to practical application, implementation of new knowledge, and/or be deliverable at scale?”
5. Impact on equity – “Would you say that the proposed research would be likely to improve equity among the sufferers from COVID-19, their carers, and in society as a whole?”

### Scoring of the proposed research questions and analysis of the results

All 79 experts who proposed three or more research options received the consolidated list of 192 questions. The process of checking and revising the questions, which was led by the MCG, and of retaining as many of 192 ideas, ensured that there is sufficient diversity in both the type of research and the area of research and that all the key areas were represented.

The 79 experts were asked to score each question according to the previously explained criteria, using an Excel sheet. If the research question was likely to satisfy the criterion, they were asked to enter the value “1”. If not, they were asked to enter the value “0”. If they did not have enough knowledge to respond, they were instructed to leave a blank cell. Finally, if they had sufficient knowledge on the topic, but were unsure, they were instructed to enter the value “0.5”, though this was generally discouraged. The reason is that the response “0.5” reduces the discriminatory power of the exercise and leads to the “regression to the mean” in the final distribution of the overall “Research Priority Scores” (RPS) [18,20-26]. The scoring was made with no additional weighting or adjustments. A total of 50 ISoGH’s experts provided scores, which were later supplemented by the scores of two more members of the MCG team, yielding 52 scores used in the priority setting exercise.

Based on the input from 52 experts, the MCG members generated intermediate “Criterion-Specific Scores” (CSS) by calculating the mean of the individual scores for each research question and each criterion received from all experts. All CSS ranged from 0 to 100%. Subsequently, the overall RPS assigned to each research question was a simple mean of all five CSS. “Average Expert Agreement” (AEA), an indicator of the average proportion of scorers that returned the most common answer for a research question, was also calculated for each research question to provide an understanding of the level of agreement among scorers. This is expressed as the frequency of the mode (ie, the most common score divided by the total number of scores), as follows:



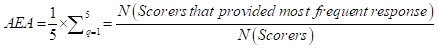



where “*5*” represents the five criteria, and “*N*” is the total number of experts who provided scores [18,20-26].

## RESULTS

**Table 1** shows the 15 highest-scoring research priorities related to addressing the burden of COVID-19 in LMICs. Among these top priorities, questions regarding vaccination were prominent: studying the barriers in access to health care systems for equitable uptake of COVID-19 vaccination was ranked 1st; studying factors that determine vaccine hesitancy was ranked 4th; development and evaluation of effective interventions to decrease vaccine hesitancy was 5th; studying the impact of the COVID-19 vaccine on vulnerable populations was 6th; identifying factors that drive COVID-19 vaccine acceptance in LMIC population was 12th; and studying how can COVID-19 vaccine delivery be improved in LMIC to ensure cold chain was 13th. This means that 6 of the top 15 questions were concerned with the issue of equitable vaccine delivery with ensured cold-chain and wide acceptance.

**Table 1:** The 15 highest-scoring research priorities related to addressing the burden of COVID-19 in LMIC*

Rank	Question	Feasibility and answerability	Potential for burden reduction	Potential for a paradigm shift	Potential for translation and implementation	Impact on equity	RPS	AEA
**1**	Studying the barriers in access to health care system for equitable uptake of COVID-19 vaccination	0.98	0.94	0.71	0.95	0.957	0.90	0.90
**2**	Identifying the most effective strategies in the management of COVID-19 globally and in LMIC?	0.83	0.92	0.81	0.90	0.83	0.86	0.85
**3**	Studying how to integrate care for COVID-19 with other essential health services in LMIC?	0.96	0.82	0.65	0.90	0.898	0.84	0.84
**4**	Studying factors that determine vaccine hesitancy in LMIC settings	1.00	0.83	0.66	0.92	0.80	0.84	0.83
**5**	Development and evaluation of effective interventions to decrease vaccine hesitancy in general population	0.95	0.90	0.70	0.88	0.77	0.84	0.83
**6**	Studying the impact of the COVID-19 vaccine on vulnerable populations in LMIC context	0.94	0.81	0.66	0.82	0.87	0.82	0.81
**7**	Assessing the needs of people infected with COVID-19 with respect to access to health care in rural areas of LMIC	0.87	0.83	0.57	0.89	0.913	0.81	0.80
**8**	Studying the leading socioeconomic determinants and consequences of the COVID-19 pandemic in LMIC using multi-faceted approach	0.85	0.81	0.73	0.83	0.84	0.81	0.80
**9**	Studying why some LMIC countries apparently have higher pediatric CFRs from COVID-19	0.90	0.85	0.72	0.77	0.81	0.81	0.81
**10**	Exploring the challenges and effective strategies for community engagement against COVID-19 in different LMIC contexts	0.90	0.82	0.72	0.82	0.78	0.81	0.80
**11**	Evaluating the effectiveness of interventions to reduce the psychological burden among health care workers in LMIC during the COVID-19 pandemic?	0.97	0.79	0.63	0.92	0.68	0.80	0.78
**12**	Identifying factors that drive COVID-19 vaccine acceptance in LMIC population	0.97	0.87	0.65	0.84	0.64	0.79	0.78
**13**	Studying how can we improve COVID-19 vaccine delivery in LMIC to ensure cold chain?	0.95	0.72	0.62	0.88	0.80	0.79	0.79
**14**	Studying how to Improve availability, access and regulations of medicines in LMIC to improve COVID-19-related outcomes	0.87	0.87	0.57	0.81	0.84	0.79	0.79
**15**	Studying how best to implement cost-effective, comprehensive and sustainable measures to limit COVID-19 transmission in LMIC?	0.80	0.87	0.72	0.82	0.74	0.79	0.79

RPS – Research Priority Score, AEA – Average Expert Agreement, LMIC – low- and middle-income countries, CFR – case fatality rates

*The priorities were ranked according to their overall “Research Priority Score” (RPS), while the last column denotes “Average Expert Agreement” (AEA).

Identification of the most effective strategies to manage COVID-19 globally and in LMICs was ranked as the 2nd highest research priority, while studying how to integrate health care for COVID-19 with other essential health services in LMICs was 3rd. Additionally, the assessment of the needs of COVID-19 patients with respect to access to health care in rural areas of LMIC was ranked 7th, and studying the leading socioeconomic determinants and consequences of the COVID-19 pandemic in LMICs using a multi-faceted approach was 8th. The remaining questions in the top 10 were: estimating paediatric case-fatality rates (CFRs) from COVID-19 in LMICs, and exploring the challenges and effective strategies for community engagement against COVID-19 in different LMIC contexts. Three more questions made it to the top 15: evaluating the effectiveness of interventions to reduce the psychological burden among health care workers (HCW) in LMICs during the COVID-19 pandemic was ranked 11th, clearly recognising the burden on HCW as a problem that is even more prominent in LMIC context; then, studying how to improve availability, access and regulation of medicines in LMIC to improve COVID-19-related outcomes was ranked 14th; and studying how best to implement cost-effective, comprehensive and sustainable measures to limit COVID-19 transmission in LMIC was 15th.

**Table 2** shows the 15 lowest-scoring research questions related to addressing the burden of COVID-19 in LMICs. The five lowest-scoring proposed research questions were “Studying the effects of most consumed beverages on COVID-19 outcomes in different LMIC settings”, “Studying how best to design and implement workshops about reading ‘the language of nature’ in educational institutions in LMICs”, “Studying the effects of global social habits (e.g., garlic and onion rich foods, using salt as gargle, etc.) to prevent the effects of COVID-19 in LMICs”, “Evaluating the importance of correct and timely defining of the status of the COVID-19 as epidemic or pandemic”, and “Exploring if Sustainable Development Goals in LMICs are still attainable by 2030”. Although all five of these questions are clearly interesting and they could be addressed through research, there was a lack of collective optimism, which was reflected mainly in low scores on the “Impact on equity” criterion for the bottom four (criterion-specific scores ranging from 0.26 to 0.30), low potential for a paradigm shift (0.26 to 0.46), burden reduction (0.29 to 0.47), and translation and implementation (0.34 to 0.50).

**Table 2:** The 15 lowest-scoring research questions related to addressing the burden of COVID-19 in LMIC*

Rank	Question	Feasibility and answerability	Potential for burden reduction	Potential for a paradigm shift	Potential for translation and implementation	Impact on equity	RPS	AEA
178	Studying how to increase caregivers' involvement in early childhood development in LMIC during COVID-19?	0.73	0.42	0.31	0.67	0.56	0.54	0.63
179	Conducting qualitative research with officials to explore the municipal role of public health	0.74	0.44	0.45	0.56	0.49	0.54	0.56
180	Evaluating the post-pandemic growth experiences among the marginalised groups	0.58	0.42	0.40	0.55	0.73	0.54	0.60
181	Evaluating if involvement of experts from LMIC improves the development of preventive strategies against COVID-19, including vaccines	0.63	0.48	0.48	0.51	0.56	0.53	0.53
182	Studying if there are unforeseen effects of the TB vaccines on COVID-19, and COVID-19 vaccines on TB	0.74	0.46	0.44	0.58	0.37	0.52	0.58
183	Studying how did the COVID-19 pandemic affect the reliability of neonatal, infant and child mortality rates in LMICs?	0.62	0.39	0.43	0.55	0.55	0.51	0.57
184	Estimating the seroprevalence of antibodies against SARS-CoV-2 in LMICs throughout the pandemic	0.71	0.53	0.54	0.48	0.28	0.51	0.59
185	Evaluating the effects of the traditional Chinese medicine on treatment of COVID-19 in LMIC	0.55	0.45	0.43	0.57	0.43	0.49	0.55
186	Evaluating the impact of COVID-19 pandemic on breastfeeding rates in LMICs	0.79	0.38	0.35	0.45	0.41	0.48	0.63
187	Evaluating the impact of COVID-19 pandemic on child marriage in LMIC	0.72	0.34	0.34	0.43	0.52	0.47	0.61
188	Exploring if sustainable development goals in LMIC are still attainable by 2030?	0.60	0.39	0.38	0.48	0.45	0.46	0.56
189	Evaluating the importance of correct and timely defining of the status of the COVID-19 as epidemic or pandemic	0.60	0.47	0.46	0.34	0.26	0.43	0.60
190	Studying the effects of global social habits (eg, garlic and onion rich foods, using salt as gargle, etc.) to prevent the effects of COVID-19 in LMICs	0.48	0.30	0.42	0.40	0.30	0.38	0.60
191	Studying how best to design and implement workshops about reading “the language of nature” in education institutions in LMIC?	0.50	0.35	0.26	0.50	0.26	0.37	0.62
192	Studying the effects of most consumed beverages on COVID-19 outcomes in different LMIC settings	0.55	0.29	0.27	0.42	0.27	0.36	0.63

RPS – Research Priority Score, AEA – Average Expert Agreement, LMIC – low- and middle-income countries, TB – tuberculosis

*The priorities were ranked according to their overall “Research Priority Score” (RPS), while the AEA column denotes “Average Expert Agreement”.

There were several research questions that ended in the 15 lowest-scoring questions because, although they scored quite high on some of the criteria, their overall score was low due to an apparent problem related to one or two specific criteria. Examples are the research questions “Evaluating the impact of COVID-19 pandemic on child marriage in LMIC”, “Evaluating the impact of COVID-19 pandemic on breastfeeding rates in LMICs”, “Evaluating the effects of the traditional Chinese medicine on treatment of COVID-19 in LMICs”, “Studying how to increase caregivers' involvement in early childhood development in LMICs during COVID-19”, and “Studying how did the COVID-19 pandemic affect the reliability of neonatal, infant, and child mortality rate estimates in LMICs”, which had low scores for the possible impact on burden reduction and/or a paradigm shift. Another example is the proposed research question “Estimating the seroprevalence of antibodies against SARS-CoV-2 in LMICs throughout the pandemic”, which had a low score related to its predicted impact on equity.

**Table 3** presents the 10 highest-scoring research questions according to the criterion “feasibility and answerability”. Particularly interesting questions on this list are those that did not make it into the top 15 research priorities (**Table 1**), but still scored very highly on this criterion alone. The examples are “Development and evaluation of effective interventions to improve health care provider’s COVID-19 knowledge and communication skills” (criterion-specific score (CSS) = 0.98, overall RPS = 0.71); then, “Studying how did the COVID-19 pandemic affect prenatal care and maternal mortality in LMICs” (CSS = 0.97, RPS = 0.72); and “Studying the role of health literacy in understanding health information regarding COVID-19” (CSS = 0.96; RPS = 0.77).

**Table 3:** The 10 highest-scoring research questions according to the criterion “Feasibility and answerability”

Rank	Question	Feasibility and answerability	Potential for burden reduction	Potential for a paradigm shift	Potential for translation and implementation	Impact on equity	RPS	AEA
1	Studying factors that determine vaccine hesitancy in LMIC settings	1.00	0.83	0.66	0.92	0.80	0.84	0.83
2	Studying the barriers in access to health care system for equitable uptake of COVID-19 vaccination	0.98	0.94	0.71	0.95	0.957	0.90	0.90
3	Development and evaluation of effective interventions to improve health care provider’s COVID-19 knowledge and communication skills	0.98	0.69	0.48	0.84	0.59	0.71	0.72
4	Identifying factors that drive COVID-19 vaccine acceptance in LMIC population	0.97	0.87	0.65	0.84	0.64	0.79	0.78
5	Studying how did the COVID-19 pandemic affect prenatal care and maternal mortality in LMIC	0.97	0.61	0.52	0.77	0.74	0.72	0.71
6	Evaluating the effectiveness of interventions to reduce the psychological burden among health care workers in LMIC during the COVID-19 pandemic	0.97	0.79	0.63	0.92	0.68	0.80	0.78
7	Studying how to integrate care for COVID-19 with other essential health services in LMIC?	0.96	0.82	0.65	0.90	0.898	0.84	0.84
8	Studying the role of health literacy in understanding health information regarding COVID-19	0.96	0.70	0.59	0.83	0.80	0.77	0.77
9	Studying how can we improve COVID-19 vaccine delivery in LMIC to ensure cold chain?	0.95	0.72	0.62	0.88	0.80	0.79	0.79
10	Development and evaluation of effective interventions to decrease vaccine hesitancy in general population	0.95	0.90	0.70	0.88	0.77	0.84	0.83

RPS – Research Priority Score, AEA – Average Expert Agreement, LMIC – low- and middle-income countries, TB – tuberculosis

**Table 4** presents the 10 highest-scoring research questions according to the criterion “potential for burden reduction”. The research questions in this table largely ranked very highly in the overall assessment, with one exception. Interestingly, the research question with the greatest collective optimism from the experts towards this particular criterion and the highest value of CSS (0.94), but which didn’t make it into the top 15 based on the overall RPS, was the question “Studying the impact of school reopenings on COVID-19 morbidity in different age groups in the general population in LMIC context”. Regardless of such a high CSS, the question was eventually ranked lower because of somewhat poorer scores for the “Potential for a paradigm shift” and “Impact on equity”.

**Table 4:** The 10 highest-scoring research questions according to the criterion “Potential for burden reduction”

Rank	Question	Feasibility and answerability	Potential for burden reduction	Potential for a paradigm shift	Potential for translation and implementation	Impact on equity	RPS	AEA
1	Studying the impact of school reopenings on COVID-19 morbidity in different age groups in the general population in LMIC context	0.86	0.94	0.59	0.74	0.64	0.76	0.69
2	Studying the barriers in access to health care system for equitable uptake of COVID-19 vaccination	0.98	0.94	0.71	0.95	0.957	0.90	0.90
3	Identifying the most effective strategies in the management of COVID-19 globally and in LMIC?	0.83	0.92	0.81	0.90	0.83	0.86	0.85
4	Development and evaluation of effective interventions to decrease vaccine hesitancy in general population	0.95	0.90	0.70	0.88	0.77	0.84	0.83
5	Identifying factors that drive COVID-19 vaccine acceptance in LMIC population	0.97	0.87	0.65	0.84	0.64	0.79	0.78
6	Studying how to Improve availability, access and regulations of medicines in LMIC to improve COVID-19-related outcomes	0.87	0.87	0.57	0.81	0.84	0.79	0.79
7	Studying how best to implement cost-effective, comprehensive and sustainable measures to limit COVID-19 transmission in LMIC?	0.80	0.87	0.72	0.82	0.74	0.79	0.79
8	Studying why some LMIC countries apparently have higher pediatric CFRs from COVID-19	0.90	0.85	0.72	0.77	0.81	0.81	0.81
9	Studying factors that determine vaccine hesitancy in LMIC settings	1.00	0.83	0.66	0.92	0.80	0.84	0.83
10	Assessing the needs of people infected with COVID-19 with respect to access to health care in rural areas of LMIC	0.87	0.83	0.57	0.89	0.913	0.81	0.80

RPS – Research Priority Score, AEA – Average Expert Agreement, LMIC – low- and middle-income countries

**Table 5** presents the 10 highest-scoring research questions according to the criterion “Potential for a paradigm shift”. The second- and third-ranked research questions on this criterion had a rather unremarkable overall RPS: “Studying the interplay of environmental and genetic factors in COVID-19 severity in LMIC” and “Studying why were some African and other LMICs relatively spared of COVID-19”. Clearly, although the experts were reserved towards those two research questions in terms of the questions satisfying other criteria, answers to those two questions could potentially change the way we see the COVID-19 pandemic in a substantial way. Following them in the fourth rank was another question that didn’t make it to the top 15 overall: “Developing large longitudinal cohorts to study long COVID-19 in LMIC settings”. Two further questions that didn’t make it into the top 15, but that could potentially lead to a paradigm shift during this pandemic were “Studying how long does protective immunity last in vaccinated people in LMIC populations” and “How should mobilising research capacity across LMICs and “South-South” collaborations best be achieved to search for an effective COVID-19 treatment”.

**Table 5:** The 10 highest-scoring research questions according to the criterion “Potential for a paradigm shift”

Rank	Question	Feasibility and answerability	Potential for burden reduction	Potential for a paradigm shift	Potential for translation and implementation	Impact on equity	RPS	AEA
1	Identifying the most effective strategies in the management of COVID-19 globally and in LMIC?	0.83	0.92	0.81	0.90	0.83	0.86	0.85
2	Studying the interplay of environmental and genetic factors in COVID-19 severity in LMIC	0.68	0.66	0.79	0.68	0.65	0.69	0.67
3	Studying why were some African and other LMIC countries relatively spared of COVID-19?	0.79	0.61	0.77	0.60	0.52	0.66	0.64
4	Developing large longitudinal cohorts to study long COVID-19 in LMIC settings	0.76	0.76	0.77	0.80	0.71	0.76	0.73
5	Studying the leading socioeconomic determinants and consequences of the COVID-19 pandemic in LMIC using multifaceted approach	0.85	0.81	0.73	0.83	0.84	0.81	0.80
6	Studying how long does protective immunity last in vaccinated people in LMIC populations	0.87	0.81	0.73	0.79	0.62	0.76	0.75
7	Studying how best to implement cost-effective, comprehensive and sustainable measures to limit COVID-19 transmission in LMIC?	0.80	0.87	0.72	0.82	0.74	0.79	0.79
8	Mobilizing research capacity across LMIC and “South-South” collaborations to search for an effective COVID-19 treatment	0.71	0.80	0.72	0.73	0.965	0.78	0.73
9	Exploring the challenges and effective strategies for community engagement against COVID-19 in different LMIC contexts	0.90	0.82	0.72	0.82	0.78	0.81	0.80
10	Studying why some LMIC countries apparently have higher pediatric CFRs from COVID-19	0.90	0.85	0.72	0.77	0.81	0.81	0.81

RPS – Research Priority Score, AEA – Average Expert Agreement, LMIC – low- and middle-income countries, CFR – case fatality rates

**Table 6** presents the 10 highest-scoring research questions according to the criterion “Potential for translation and implementation” of the proposed research. Two of these questions were very high on the overall list – not among the top 15, but in the top 20. Those were “Prospectively evaluating the long-term effects of COVID-19 on mental health and well-being in different age groups” and “Studying how best to plan human resource, training, and priority-setting needs during the COVID-19 pandemic in LMICs”. The two other questions that did particularly well on this criterion were “Developing very low-cost diagnostic kits for COVID-19 for use in LMICs” and “Studying the feasibility of developing data-driven disease surveillance systems in LMICs”. Clearly, both ideas would have the potential to make a large positive difference to the pandemic in LMICs if they were more feasible in low-resource settings.

**Table 6:** The 10 highest-scoring research questions according to the criterion “Potential for translation and implementation”

Rank	Question	Feasibility and answerability	Potential for burden reduction	Potential for a paradigm shift	Potential for translation and implementation	Impact on equity	RPS	AEA
1	Studying the barriers in access to health care system for equitable uptake of COVID-19 vaccination	0.98	0.94	0.71	0.95	0.95	0.90	0.90
2	Evaluating the effectiveness of interventions to reduce the psychological burden among health care workers in LMIC during the COVID-19 pandemic?	0.97	0.79	0.63	0.92	0.68	0.80	0.78
3	Studying factors that determine vaccine hesitancy in LMIC settings	1.00	0.83	0.66	0.92	0.80	0.84	0.83
4	Identifying the most effective strategies in the management of COVID-19 globally and in LMIC?	0.83	0.92	0.81	0.90	0.83	0.86	0.85
5	Studying how to integrate care for COVID-19 with other essential health services in LMIC?	0.96	0.82	0.65	0.90	0.89	0.84	0.84
6	Developing very low-cost diagnostic kits for COVID-19 for use in LMIC	0.83	0.77	0.57	0.90	0.82	0.78	0.76
7	Studying the feasibility of developing data-driven disease surveillance systems in LMIC	0.87	0.64	0.67	0.89	0.66	0.75	0.74
8	Prospectively evaluating the long-term effects of COVID-19 on mental health and well-being in different age groups	0.91	0.69	0.68	0.89	0.76	0.79	0.78
9	Assessing the needs of people infected with COVID-19 with respect to access to health care in rural areas of LMIC	0.87	0.83	0.57	0.89	0.91	0.81	0.80
10	Studying how best to plan human resource, training and priority-setting needs during the COVID-19 pandemic in LMIC?	0.91	0.75	0.56	0.89	0.82	0.79	0.78

RPS – Research Priority Score, AEA – Average Expert Agreement, LMIC – low- and middle-income countries

**Table 7** presents the 10 highest-scoring research questions according to the criterion “Impact on equity”. The most highly ranked was the proposed research question “Mobilising research capacity across LMICs and ‘South-South’ collaborations to search for an effective COVID-19 treatment”, which was not among the top 15 questions overall. Other important research questions that could improve equity in LMICs, but didn’t make the top 15 priorities overall, included “Which digital technologies can be used to address inequities and improve health care access in LMICs during the pandemic?”; “Studying the emerging effects of COVID-19 on marginalised and vulnerable women and girls”; “Descriptive research on the social, economic, and health impacts of COVID-19 pandemic on women and other vulnerable groups and equity in LMICs”; “Developing models to reduce inequities in health and education that resulted from the COVID-19 pandemic in LMICs”; and “Studying characteristics and capacities of the primary health care (PHC) in the rural clinics to provide health care for people with COVID-19”.

**Table 7:** The 10 highest-scoring research questions according to the criterion “Impact on equity”

Rank	Question	Feasibility and answerability	Potential for burden reduction	Potential for a paradigm shift	Potential for translation and implementation	Impact on equity	RPS	AEA
1	Mobilizing research capacity across LMIC and “South-South” collaborations to search for an effective COVID-19 treatment	0.71	0.80	0.72	0.73	0.965	0.78	0.73
2	Studying the barriers in access to health care system for equitable uptake of COVID-19 vaccination	0.98	0.94	0.71	0.95	0.957	0.90	0.90
3	Which digital technologies can be used to address inequities and improve health care access in LMIC during the pandemic?	0.83	0.78	0.57	0.81	0.941	0.79	0.78
4	Studying the emerging effects of COVID-19 on marginalised and vulnerable women and girls	0.92	0.63	0.63	0.80	0.933	0.78	0.76
5	Assessing the needs of people infected with COVID-19 with respect to access to health care in rural areas of LMIC	0.87	0.83	0.57	0.89	0.913	0.81	0.80
6	Studying how to integrate care for COVID-19 with other essential health services in LMIC?	0.96	0.82	0.65	0.90	0.898	0.84	0.84
7	Descriptive research on the social, economic, and health impacts of COVID-19 pandemic on women and other vulnerable groups and equity in LMIC	0.93	0.57	0.54	0.67	0.88	0.72	0.71
8	Developing models to reduce inequities in health and education that resulted from the COVID-19 pandemic in LMIC	0.81	0.76	0.60	0.80	0.87	0.77	0.74
9	Studying the impact of the COVID-19 vaccine on vulnerable populations in LMIC context	0.94	0.81	0.66	0.82	0.87	0.82	0.81
10	Studying characteristics and capacities of the PHC in the rural clinics to provide health care for people with COVID-19	0.90	0.81	0.58	0.80	0.86	0.79	0.78

RPS – Research Priority Score, AEA – Average Expert Agreement, LMIC – low- and middle-income countries

## DISCUSSION

### Statement of principal findings

As the COVID-19 pandemic continues, it is increasingly important to identify research priorities for LMICs, given the scarce resources and other health systems challenges in these contexts. In this exercise, four of the top 10 questions (ranked 1,4,5,6) prioritised improving availability, access, and uptake of COVID-19 vaccines, including limiting vaccine hesitancy. Four of the top 10 questions (ranked 1,3,7,10) focused on improving access to, and utility of, health care services and systems in an LMIC context, including maximising access to vaccines, ensuring rural communities can access care, and developing community engagement strategies for COVID-19. Improving care for COVID-19 in LMICs (2), identifying socioeconomic determinants of poor outcomes (8), and studying why LMICs have higher paediatric CFRs (9) were also prioritised.

Studying the barriers and access to health care systems for equitable uptake of COVID-19 vaccines had an RPS of 0.90 (compared to 0.86 for the second-highest ranked research priority). There was high agreement on the importance of this research question (AEA = 0.90). Given the increased risk of mutations in populations who are less vaccinated, the existing barriers to (non-COVID-19) vaccines in LMICs, and the exacerbation of both problems during COVID-19, it is not surprising that this is an absolute priority.

It is interesting that reducing vaccine hesitancy was rated highly in two questions (ranked 4, 5). While vaccine hesitancy is a prominent barrier to vaccination uptake in many HICs, there have been limited studies of this phenomenon in LMICs [28]. Levels of vaccine hesitancy (in both HICs and LMICs) may be different regarding the COVID-19 vaccine as well as other routine immunisations, and the reasons for hesitancy are likely to differ between HICs and LMICs; indeed, lack of access may be conflated with hesitancy in LMICs. A study conducted in 2019, before the COVID-19 pandemic, described vaccine hesitancy in Bangladesh, China, Ethiopia, Guatemala, and India. It showed a lack of association between education and vaccine hesitancy, which contrasts with the results of studies in HIC [29]. Another study in Northwest Nigeria examined attitudes towards the COVID-19 vaccine among health science students [30]. The study found that older age, instructions by the heads of institutions, trust in government, and readiness to pay for the vaccine positively impacted vaccine acceptance [30]. A third study showed that willingness to take the vaccine is higher among people living in LMICs than in the USA and Russia (80.3%, 64.6%, and 30.4% of the population willing to take the vaccine, respectively) [31].

Government responses to the COVID-19 pandemic have been very different. The scope of the differences in responses as well as individual countries’ responses (eg, denial of existence in Tanzania during the beginning of the pandemic or the introduction of new, wide-sweeping, and restrictive laws) could affect the public’s trust in government and willingness to take vaccines.

### Strengths and limitations

CHNRI is a research priority exercise conducted online, independently, and where individual responses are not accessible by peers. This facilitates more creative and bold ideas (by lessening the fear of being associated with a “bad idea”) and enables equal weighting of experts. In other exercises, more junior experts may feel obliged to mirror ideas posed by those who are senior to them. CHNRI also enables broad participation due to its online and flexible nature.

This research prioritisation exercise relied on the participation of experts active in the ISoGH. As the COVID-19 pandemic and its field of study are relatively new, we sought to invite a broad range of participation from those who have experience and expertise in global health generally, rather than limit the exercise to experts on COVID-19, who may not be as familiar with LMIC contexts. It is possible there are some differences between those who did and did not participate, possibly resulting in some selection bias. However, for the CHNRI prioritization exercise to be useful, it is not critical that the 52 scorers are representative of the entire ISoGH membership; instead, they should be representative of the entire global health research community. This is an important difference because the hundreds of members of ISoGH are also a small and self-selected sample of a much larger global health research community, so they, too, are unlikely to be representative. The 52 scorers themselves are then a self-selected sub-sample of the ISoGH members.

Therefore, the question of possible selection bias is not addressed by ensuring that the 52 scorers are representative of the entire ISoGH membership, but rather of the entire global health research community. Since such representativeness cannot be easily ensured through any approach, the CHNRI method relies on the useful properties of crowdsourcing. It gives the best results when the scorers’ individual views are their private views, and when there is a large diversity and independence of opinions. Previous empirical research established that the rankings of research questions by independent individuals with some private knowledge of the subject become stable and robust after about 40-45 scorers take part, after which the ranks do not change much with the addition of further scorers [23,25]. We empirically showed that such a process provides surprisingly robust results and that saturation of opinions and stabilization of scores occurs quite quickly if those conditions are met, and that a sample size of 52 informed participants should be sufficient [23,25].

This is why the crowdsourcing approach to setting research priorities works quite well and is not too sensitive to the composition of the group, as long as they have independent knowledge and are well-minded and motivated to conduct the exercise. Therefore, the key to an informative CHNRI prioritization process is to ensure at least 40-45 scorers with sufficient qualifications and diversity of backgrounds, expertise, and opinion. With 52 scorers, each individual scorer contributes less than 2% to the overall rankings of proposed research ideas. Therefore, the resulting priorities are truly collective priorities, rather than those of any specific participant.

Despite having a large number of research priorities to score, over 70% of participants who submitted research questions also completed the scoring. This signifies high levels of engagement among those participants who chose to take part in the exercise. These priorities reflect a wide diversity of ISoGH members, most of whom were either stationed in an LMIC or had close ties to the LMIC setting. This is why this study delivers an opportunity to capture the real-life priorities and therefore be more attuned to local conditions than other study designs might.

The COVID-19 pandemic, resulting challenges, and practised interventions are rapidly evolving. Although this exercise was completed within six months, it is possible that shifts in evidence, development of new interventions or containment measures, and disease epidemiology and burden may have rapid shifts and advancements. It is, therefore, necessary to consider the timeline of this exercise when interpreting the results and priorities in the future.

### Interpretation in light of published literature

Improving access to, and acceptability of vaccines was firmly prioritised, with four research questions relating to these appearing in the top 10. These scored especially high in the feasibility/acceptability and potential for translation and implementation criteria. There have been a host of articles published, both in peer-reviewed journals and in newspapers calling for increased vaccine equity [32,33]. As the pandemic has shown, all countries are vulnerable when one is unprotected; the emergence of the Omicron variant, which spread rapidly after its discovery in South Africa, provides a clear illustration of our interdependence [19].

Past epidemics, such as Ebola, have showcased the fragility of health systems in LMICs [34]. Across the world, health systems have been overstretched due to increased ICU admissions combined with staff absences [35]. The focus on the continuation of health service delivery, improving equity in delivery, and integrating care for those infected with COVID-19 into health systems is therefore unsurprising.

### Implications for policy, practice, and research

This study represents an attempt to generate and systematically score research priorities to address COVID-19 across LMICs. The highest-scoring research priority identified was to study barriers to accessing the health system, specifically, those related to equitable uptake of the COVID-19 vaccine. As described earlier, the global population is interdependent; no person or country is safe until everyone has some protection. Globally, almost 63% of the population has received at least one dose of a COVID-19 vaccine; however, in Africa, this proportion drops to 15% [36].

Covax, which is co-led by the Global Alliance for Vaccines and Immunization (GAVI), the Coalition for Epidemic Preparedness Innovations (CEPI), and the World Health Organization (WHO) aim to accelerate vaccine distribution in LMICs. COVAX estimated that introducing the vaccine globally will prevent losses of approximately $375 billion USD [37]. In addition to COVAX, there have been calls for the World Trade Organization (WTO) to waive intellectual property (IP) rights on the COVID-19 vaccines; however, there is a debate on whether this would promote increased production as some manufacturers may not be sufficiently equipped.

It is quite possible that LMICs may have some of their research priorities defined and supported already, so further investigation into how they align with the research priorities identified in this research paper would possibly be interesting. However, it is difficult to generalize the priorities for the entire LMIC part of the world, as it consists of many countries which have different priorities. Many of them do not really have a defined list of priorities at all, but rather respond to the most pressing and urgent public health needs using their scarce resources, so this exercise may assist them in further planning.

Investment in health systems strengthening, including identifying LMIC-specific best practice strategies for managing COVID-19 and integrating care for COVID-19 patients into other health services are imperative. Community Health Workers/Volunteers (CHW/CHV), employed in a number of LMICs, serve their communities through door-to-door visits, but have been impeded due to the risk of infection. Households residing in LMICs have had disruptions to their employment, and the resulting impact on livelihoods may have decreased the affordability of health services [38]. It is imperative that global cooperation exists to ensure effective and targeted research and programming, as it will ultimately improve the global COVID-19 response by reducing mortality and morbidity, and by providing a pathway to economic recovery.

## Additional material


Online Supplementary Document
