# Supplementary material for: Research priorities to reduce the impact of COVID-19 in low- and middle-income countries
Source: J Glob Health. 2022 Apr 15;12:09003. doi: 10.7189/jogh.12.09003 (PMC9010705; doi:10.7189/jogh.12.09003)
Supplement: Online Supplementary Document [file jogh-12-09003-s001.pdf]

Supplementary Table 1. The full list of research questions with scores and ranks.

Rank	Research question	Feasibility and Answerability	Potential for Burden Reduction	Potential for a Paradigm Shift	Potential For Translation and Implementation	Impact on Equity	RPS	AEA
1	Evaluating the importance of correct and timely defining of the status of the COVID-19 as epidemic or pandemic	0.60	0.47	0.46	0.34	0.26	0.43	0.60
2	Studying why some LMIC countries apparently have higher pediatric CFRs from COVID-19	0.90	0.85	0.72	0.77	0.81	0.81	0.81
3	Performing a comparative study of COVID-19 morbidity and mortality across many LMIC countries	0.88	0.57	0.66	0.61	0.66	0.68	0.66
4	Using PATHS tool to understand the mortality patterns of COVID-19 in LMIC	0.81	0.65	0.71	0.69	0.65	0.70	0.66
5	Comparing COVID-19 perception. prevention and treatment across LMIC countries	0.92	0.58	0.71	0.67	0.65	0.71	0.70
6	Studying why were some African and other LMIC countries relatively spared of COVID-19?	0.79	0.61	0.77	0.60	0.52	0.66	0.64
7	Identifying the most effective strategies in the management of COVID-19 globally and in LMIC?	0.83	0.92	0.81	0.90	0.83	0.86	0.85
8	Assessing the best way for LMIC to adopt global initiatives and management of pandemics?	0.56	0.69	0.57	0.62	0.68	0.62	0.60
9	Evaluating the effect of full vaccination of the persons in LMIC on COVID-19-related outcomes	0.89	0.77	0.60	0.75	0.75	0.75	0.74
10	Identifying optimal ways to model country-specific determinants of COVID-19 spread	0.70	0.76	0.61	0.68	0.60	0.67	0.65
11	Estimating the seroprevalence of antibodies against SARS-CoV-2 in LMICs throughout the pandemic	0.71	0.53	0.54	0.48	0.28	0.51	0.59
12	Studying the interplay of environmental and genetic factors in COVID-19 severity in LMIC	0.68	0.66	0.79	0.68	0.65	0.69	0.67
13	Studying the transmission pattern across different COVID-19 variants in LMIC	0.73	0.68	0.66	0.67	0.52	0.65	0.65
14	Studying the effects of contact-tracing in mitigating the spread of COVID-19 in LMIC	0.82	0.72	0.52	0.77	0.52	0.67	0.66
15	Identifying ways to improve research collaboration within and between LMIC and HIC during the times of COVID-19	0.83	0.58	0.58	0.68	0.66	0.66	0.65

16	Studying how did the COVID-19 pandemic affect the cost of medicines in LMIC?	0.91	0.44	0.43	0.44	0.76	0.59	0.66
17	Identifying the key obstacles to lowering the cost of medicines for LMIC during COVID-19 pandemic?	0.88	0.61	0.49	0.65	0.76	0.68	0.68
18	Studying how can digital interventions address the secondary outcomes of the COVID-19 pandemic in LMIC?	0.78	0.78	0.62	0.78	0.73	0.74	0.72
19	Studying how can we assess and improve the teleconsultation services in LMIC during the COVID-19 pandemic?	0.87	0.81	0.58	0.86	0.84	0.79	0.78
20	Implementation research on the alternate modes of COVID-19-related health delivery in LMIC beyond telemedicine reliance	0.77	0.75	0.62	0.69	0.75	0.72	0.69
21	Developing fast surveillance and screening methods at the borders using AI for the fastest control of new cases?	0.59	0.64	0.51	0.57	0.42	0.55	0.56
22	Studying how to develop global digital platforms for supporting LMIC health care providers and organisations during the pandemic?	0.75	0.63	0.64	0.79	0.69	0.70	0.68
23	Evaluating what MNCH interventions can be provided online safely during the COVID-19 pandemic?	0.80	0.59	0.51	0.80	0.63	0.67	0.66
24	What is the cost-effectiveness of the online MNCH interventions during the COVID-19 pandemic?	0.82	0.61	0.49	0.72	0.63	0.65	0.64
25	Which digital technologies can be used to address inequities and improve health care access in LMIC during the pandemic?	0.83	0.78	0.57	0.81	0.941	0.79	0.78
26	Studying how can we increase the digital health literacy of community and care providers in LMIC during the COVID-19 pandemic?	0.88	0.77	0.60	0.78	0.78	0.76	0.76
27	Studying the long term impact of the COVID-19 pandemic on human resources in health	0.85	0.65	0.54	0.73	0.66	0.69	0.67
28	Assessing the acceptability and readiness for online training among care providers in LMIC	0.95	0.59	0.50	0.73	0.61	0.67	0.66
29	Studying the perception of healthcare workers about the benefits and safety of the COVID-19 vaccines	0.86	0.51	0.50	0.63	0.46	0.59	0.59
30	Studying how best to design and implement healthier lifestyle workshops in education institutions in LMIC?	0.82	0.51	0.39	0.73	0.40	0.57	0.65
31	Studying how best to design and implement workshops about reading "the language of nature" in education institutions in LMIC?	0.50	0.35	0.26	0.50	0.26	0.37	0.62
32	Studying how best to design and organise forums to learn from first-hand. "street/community-level" experience in LMIC?	0.77	0.61	0.49	0.66	0.62	0.63	0.60
33	Studying how best to plan human resource. training and priority-setting needs during the COVID-19 pandemic in LMIC?	0.91	0.75	0.56	0.89	0.82	0.79	0.78
34	Studying how to Improve availability. access and regulations of medicines in LMIC to improve COVID-19-related outcomes	0.87	0.87	0.57	0.81	0.84	0.79	0.79

35	Exploring opportunities to repurpose the existing affordable medications to improve COVID-related outcomes in LMIC?	0.82	0.78	0.56	0.69	0.84	0.74	0.71
36	Conducting qualitative research with officials to explore the municipal role of public health	0.74	0.44	0.45	0.56	0.49	0.54	0.56
37	Studying characteristics and capacities of the PHC in the rural clinics to provide health care for people with COVID-19	0.90	0.81	0.58	0.80	0.86	0.79	0.78
38	Exploring the relationship between key primary care metrics and COVID-19 public health outcomes in LMIC	0.84	0.68	0.61	0.81	0.66	0.72	0.72
39	Studying if we can improve COVID-19 outcomes in LMIC by reimagining healthcare delivery	0.60	0.68	0.59	0.63	0.63	0.63	0.61
40	Studying how does the existing infrastructure affect COVID-19-related outcomes in LMICs?	0.73	0.73	0.53	0.68	0.71	0.68	0.67
41	Studying how did factors related to public health governance affect policy making during COVID-19 in LMICs?	0.78	0.66	0.56	0.65	0.64	0.66	0.65
42	Studying how to integrate care for COVID-19 with other essential health services in LMIC?	0.96	0.82	0.65	0.90	0.898	0.84	0.84
43	Studying how best to maintain the effectiveness of political. economic and health-care systems during the COVID-19 pandemic?	0.52	0.70	0.49	0.63	0.64	0.60	0.59
44	Studying how best to implement cost-effective. comprehensive and sustainable measures to limit COVID-19 transmission in LMIC?	0.80	0.87	0.72	0.82	0.74	0.79	0.79
45	Studying how best to improve public health capability for surveillance of infectious diseases?	0.90	0.74	0.61	0.88	0.64	0.75	0.74
46	Documenting how did health systems respond to COVID-19 in LMIC?	0.89	0.54	0.56	0.73	0.65	0.68	0.67
47	Studying if we can use lessons from COVID-19's impact on access to health care and essential medicines to improve service delivery?	0.90	0.59	0.61	0.76	0.78	0.73	0.71
48	Identifying health system reforms required for the cost-effective management of COVID in LMICs	0.74	0.70	0.60	0.77	0.66	0.70	0.67
49	Documenting the fragility of health systems and services in LMIC during COVID-19 to improve resilience	0.78	0.61	0.49	0.62	0.64	0.63	0.63
50	Studying readiness and likelihood of health systems affected by the COVID-19 pandemic to recover from it	0.68	0.58	0.48	0.71	0.56	0.60	0.58
51	Identifying factors associated with effective resilience against COVID-19 and its collateral damage using socioecological framework.	0.82	0.55	0.58	0.61	0.67	0.64	0.64
52	Studying how can we strengthen the health systems in the context of COVID-19 in LMIC through policy and practice?	0.77	0.71	0.62	0.76	0.73	0.72	0.71
53	Evaluating the politics of vaccine management and rollout plans for COVID-19 in LMIC	0.85	0.71	0.66	0.76	0.74	0.74	0.74

54	Studying how can we improve COVID-19 vaccine delivery in LMIC to ensure cold chain?	0.95	0.72	0.62	0.88	0.80	0.79	0.79
55	Evaluating the vaccine coverage and uptake in African and other LMIC compared to the set goals	0.89	0.61	0.57	0.71	0.80	0.72	0.70
56	Evaluating the impact of COVID-19 pandemic on breastfeeding rates in LMICs	0.79	0.38	0.35	0.45	0.41	0.48	0.63
57	Evaluating the impact of COVID-19 pandemic on child mortality in LMIC	0.94	0.58	0.51	0.67	0.72	0.68	0.68
58	Evaluating the impact of COVID-19 pandemic on children feeding practices in LMIC	0.79	0.47	0.40	0.56	0.53	0.55	0.59
59	Evaluating the impact of COVID-19 pandemic on child marriage in LMIC	0.72	0.34	0.34	0.43	0.52	0.47	0.61
60	Studying the leading socioeconomic determinants and consequences of the COVID-19 pandemic in LMIC using multifaceted approach	0.85	0.81	0.73	0.83	0.84	0.81	0.80
61	Exploring if it is possible to use previous epidemics and pandemics to create effective policies in response to COVID-19 in LMICs	0.76	0.64	0.47	0.58	0.48	0.58	0.58
62	Exploring if sustainable development goals in LMIC are still attainable by 2030?	0.60	0.39	0.38	0.48	0.45	0.46	0.56
63	Evaluating the impact of COVID-19 pandemic on people living with HIV/AIDS in LMIC	0.93	0.61	0.42	0.77	0.78	0.70	0.72
64	Studying how to increase caregivers' involvement in early childhood development in LMIC during COVID-19?	0.73	0.42	0.31	0.67	0.56	0.54	0.63
65	Evaluating the impact of COVID-19 pandemic on child health and well-being in LMIC	0.93	0.72	0.57	0.76	0.75	0.75	0.74
66	Studying the effects of COVID-19 on the accessibility and utilisation of family planning and maternal health care services in LMIC	0.89	0.49	0.43	0.71	0.70	0.64	0.66
67	Evaluating the impact of COVID-19 on prematurity rates and exploring the effective care in the community	0.80	0.50	0.48	0.58	0.59	0.59	0.58
68	Developing self-tools for integrated pregnancy care services during COVID-19 in LMIC	0.89	0.51	0.47	0.83	0.64	0.67	0.66
69	Studying the impact of the COVID-19 pandemic on mental health vulnerability in LMICs	0.89	0.76	0.64	0.87	0.77	0.79	0.78
70	Evaluating the effectiveness of interventions to reduce the psychological burden among healthcare workers in LMIC during the COVID-19 pandemic?	0.97	0.79	0.63	0.92	0.68	0.80	0.78
71	Studying how did COVID-19 affect suicide rates in LMICs	0.73	0.49	0.41	0.64	0.58	0.57	0.58
72	Studying the effect of COVID-19 pandemic on the supply of and demand for psychiatric services in LMICs	0.86	0.60	0.43	0.83	0.65	0.67	0.67
73	Studying the impact of COVID-19 on mental health in populations with pre-existing mental disorders	0.90	0.64	0.47	0.79	0.68	0.70	0.69

74	Prospectively evaluating the long-term effects of COVID-19 on mental health and well-being in different age groups	0.91	0.69	0.68	0.89	0.76	0.79	0.78
75	Evaluating interventions that support the resilience of mental health in different age groups in LMICs during COVID-19 pandemic	0.85	0.69	0.58	0.84	0.73	0.74	0.72
76	Conducting an e-mental health randomised intervention trial by health workers in LMIC during the COVID-19 pandemic	0.74	0.65	0.53	0.67	0.56	0.63	0.60
77	Documenting the effective coping mechanisms of the general population in the COVID-19 pandemic	0.76	0.61	0.56	0.69	0.60	0.64	0.63
78	Studying the individual. group or systemic/social interventions tailored to people's coping mechanisms to manage psychosocial problems caused by the COVID-19 pandemic in LMICs	0.71	0.51	0.52	0.67	0.60	0.60	0.57
79	Studying how did the COVID-19 pandemic affect capacity and results in management of non-communicable diseases in LMIC?	0.89	0.56	0.53	0.72	0.62	0.66	0.66
80	Studying how did the COVID-19 pandemic affect exposure to risk factors for non-communicable diseases in LMIC?	0.88	0.64	0.68	0.78	0.72	0.74	0.73
81	Studying how did the COVID-19 pandemic affect prenatal care and maternal mortality in LMIC?	0.97	0.61	0.52	0.77	0.74	0.72	0.71
82	Evaluating and comparing the impact of COVID-19 pandemic in the rural versus urban areas in LMIC	0.90	0.63	0.53	0.72	0.80	0.72	0.70
83	Studying how did the COVID-19 pandemic affect the reliability of neonatal. infant and child mortality rates in LMICs?	0.62	0.39	0.43	0.55	0.55	0.51	0.57
84	Policy research on the most efficient models of technology transfer. including vaccines. between LMIC and HIC	0.84	0.77	0.64	0.84	0.81	0.78	0.77
85	Studying the feasibility of developing data-driven disease surveillance systems in LMIC	0.87	0.64	0.67	0.89	0.66	0.75	0.74
86	Creating national-based geo-referenced. health-related data collection systems linked with nationwide clinical data. communications and decision-making processes	0.73	0.73	0.71	0.71	0.76	0.73	0.71
87	Studying effective approaches to enhance analytical tools. diagnostics and regulatory capabilities	0.57	0.69	0.56	0.67	0.58	0.61	0.58
88	Studying "micro-ecology" of the respiratory tract to develop novel molecular diagnostics for rapid co-pathogens identification in COVID-19 patients	0.71	0.70	0.64	0.70	0.42	0.63	0.61
89	Developing innovative services in LMIC to offer case management to COVID-19 patients	0.67	0.79	0.50	0.70	0.76	0.68	0.66
90	Developing very low-cost diagnostic kits for COVID-19 for use in LMIC	0.83	0.77	0.57	0.90	0.82	0.78	0.76
91	Improving access to technologies to support capacity of public health response to COVID-19 pandemic in LMIC	0.79	0.72	0.63	0.77	0.70	0.72	0.71

92	Studying the applicability of the transdisciplinary One Health paradigm for epidemic preparedness and response in LMIC	0.72	0.64	0.64	0.60	0.64	0.65	0.61
93	Develop innovations to catch up with strategies for preventive interventions against the other infectious diseases in LMIC (e.g.. HIV. malaria. TB)	0.79	0.56	0.48	0.78	0.57	0.64	0.62
94	Evaluating if involvement of experts from LMIC improves the development of preventive strategies against COVID-19. including vaccines	0.63	0.48	0.48	0.51	0.56	0.53	0.53
95	Evaluating the effectiveness of social distancing as a response to COVID-19 in different LMIC settings	0.78	0.61	0.45	0.65	0.46	0.59	0.62
96	Holistic research on the impact of movement restrictions (health-related. social and economic) during COVID-19 pandemic in LMIC context	0.84	0.62	0.55	0.66	0.50	0.63	0.61
97	Holistic research on the impact of lockdowns (health-related. social and economic) during COVID-19 pandemic in LMIC context	0.85	0.66	0.58	0.71	0.54	0.67	0.64
98	Studying the impact of school reopenings on COVID-19 morbidity in different age groups in the general population in LMIC context	0.86	0.94	0.59	0.74	0.64	0.76	0.69
99	Studying the impact of mask wearing during the COVID-19 pandemic in LMIC context	0.82	0.70	0.50	0.68	0.53	0.65	0.63
100	Studying the effects of global social habits (e.g.. garlic and onion rich foods. using salt as gargle. etc.) to prevent the effects of COVID-19 in LMICs	0.48	0.30	0.42	0.40	0.30	0.38	0.60
101	Studying the level of awareness of the population about appropriate effective measures in containing the spread of COVID-19 in LMIC context	0.88	0.70	0.59	0.72	0.67	0.71	0.70
102	Studying the scalable modifiable preventive factors that can be enhanced in LMIC populations to reduce the COVID transmission (e.g.. being outdoors. healthy lifestyle)?	0.74	0.67	0.65	0.69	0.52	0.65	0.64
103	Exploring the key challenges and obstacles of adhering to COVID-19 pandemic health protocols and measures in LMIC context	0.89	0.74	0.61	0.83	0.64	0.74	0.73
104	Studying the impact of the government communication strategy of COVID-19 on its policy acceptance and COVID-19 rates among LMICs	0.84	0.69	0.51	0.73	0.60	0.67	0.66
105	Exploring the challenges and effective strategies for community engagement against COVID-19 in different LMIC contexts	0.90	0.82	0.72	0.82	0.78	0.81	0.80
106	Studying the barriers in access to healthcare system for equitable uptake of COVID-19 vaccination	0.98	0.94	0.71	0.95	0.957	0.90	0.90
107	Assessing the increases in inequality in child health within LMICs as a result of the COVID-19 pandemic	0.81	0.52	0.50	0.76	0.76	0.67	0.66
108	Studying the relationship between socioeconomic status/occupation and the adherence with prevention/treatment protocols among the infected in LMIC	0.87	0.61	0.47	0.69	0.72	0.67	0.68

109	Assessing the needs of people infected with COVID-19 with respect to access to health care in rural areas of LMIC	0.87	0.83	0.57	0.89	0.913	0.81	0.80
110	Developing models to ensure more global equity in access to the COVID-19 vaccines for LMIC in a sustainable manner	0.76	0.80	0.62	0.74	0.85	0.75	0.75
111	Developing models to ensure more equity in access to the COVID-19 vaccines within individual LMIC in a sustainable manner	0.78	0.81	0.62	0.75	0.84	0.76	0.74
112	Developing approaches to rapid modification of conditions with aim to achieve more equitable prevention of COVID-19 across LMIC societies	0.51	0.63	0.49	0.53	0.68	0.57	0.57
113	Developing models to accelerate global COVID-19 response for LMIC. especially equitable vaccination and recovery efforts	0.76	0.81	0.68	0.76	0.84	0.77	0.75
114	Developing models to reduce inequities in health and education that resulted from the COVID-19 pandemic in LMIC	0.81	0.76	0.60	0.80	0.87	0.77	0.74
115	Studying how COVID-19 exposed and deepened existing disparities in terms of gender. income. and ethnicity in LMIC	0.86	0.59	0.60	0.72	0.79	0.71	0.70
116	Development a science-based guidelines for vaccine distribution globally. e.g. LMIC coverage vs. HIC boosters	0.78	0.81	0.58	0.78	0.79	0.75	0.74
117	Studying the effectiveness and resilience of the current food supply chain and food security system during nation lockdowns in LMICs?	0.72	0.60	0.45	0.67	0.79	0.65	0.65
118	Studying the effects of COVID-19 mitigation measures on food security. livelihoods and nutrition in disaster-prone regions	0.76	0.60	0.44	0.66	0.77	0.65	0.65
119	Studying how did hard-to-reach communities mitigate against risks of getting infected with COVID-19 in LMIC settings	0.77	0.56	0.50	0.55	0.66	0.61	0.59
120	Studying the impact of the COVID-19 vaccine on vulnerable populations in LMIC context	0.94	0.81	0.66	0.82	0.87	0.82	0.81
121	Setting research priorities on social. economic and healthcare dilemmas among the transgender community during the COVID-19 pandemic	0.70	0.44	0.42	0.47	0.77	0.56	0.60
122	Evaluating the post-pandemic growth experiences among the marginalised groups	0.58	0.42	0.40	0.55	0.73	0.54	0.60
123	Adopting qualitative and mixed-methods research to provide a diversity of perspectives and plurality of expertise necessary to understand vulnerable groups during the COVID-19 pandemic	0.70	0.55	0.68	0.61	0.78	0.66	0.65
124	Studying the emerging effects of COVID-19 on marginalised and vulnerable women and girls	0.92	0.63	0.63	0.80	0.933	0.78	0.76
125	Studying the impact of COVID-19 and climate change on sexual and reproductive health and gender-based violence	0.63	0.41	0.40	0.57	0.68	0.54	0.61
126	Studying the effectiveness of measures to prevent harm for the vulnerable populations from a future pandemic	0.64	0.56	0.51	0.67	0.76	0.63	0.60

127	Gamifying risk communication and community engagement on COVID-19 to penetrate hard-to-reach communities in LMIC and prevent misinformation	0.75	0.60	0.54	0.73	0.64	0.65	0.64
128	Developing and evaluating the concept of co-contributors to mitigate the impact of COVID-19. especially among the poor and hard-to-reach communities in LMIC	0.63	0.61	0.60	0.53	0.72	0.62	0.57
129	A systematic investigation of the patterns of access to appropriate health care for COVID-10 across the society in LMICs	0.90	0.72	0.65	0.74	0.80	0.76	0.76
130	Descriptive research on the social. economic. and health impacts of COVID-19 pandemic on women and other vulnerable groups and equity in LMIC	0.93	0.57	0.54	0.67	0.88	0.72	0.71
131	Assessing the capacity of LMIC to develop vaccines and therapeutics for COVID-19 on their own	0.76	0.70	0.61	0.65	0.73	0.69	0.67
132	Health economics research to develop rapid intervention mechanisms for HIC to assist LMIC in pandemic response	0.72	0.60	0.50	0.67	0.66	0.63	0.61
133	Studying conditional cash transfer programmes to improve COVID-19 vaccine uptake in remote and hard-to-reach areas in LMIC	0.74	0.60	0.51	0.69	0.81	0.67	0.66
134	Health economics research on the Impact of COVID-19 on international health aid and government health expenditures in LMIC	0.73	0.51	0.40	0.61	0.61	0.57	0.58
135	Studying how do lifestyle patterns affect COVID-19-related outcomes in LMIC?	0.79	0.73	0.70	0.78	0.64	0.73	0.71
136	Studying the role of health literacy in understanding health information regarding COVID-19	0.96	0.70	0.59	0.83	0.80	0.77	0.77
137	Studying the effects of diet on COVID-19 outcomes in different LMIC settings	0.74	0.53	0.50	0.64	0.55	0.59	0.59
138	Studying the effects of most consumed beverages on COVID-19 outcomes in different LMIC settings	0.55	0.29	0.27	0.42	0.27	0.36	0.63
139	Documenting and analysing the responses of faith-based organisations and civil societies during the COVID-19 pandemic in LMIC context	0.80	0.52	0.41	0.57	0.55	0.57	0.60
140	Comparing the success of faith-based vaccine clinics and traditional medical clinic sites in reaching LMIC communities	0.76	0.47	0.45	0.64	0.48	0.56	0.58
141	Qualitative research to examine the preparedness of communities in LMICs for the prevention and control of COVID-19 infection	0.79	0.62	0.53	0.69	0.66	0.66	0.65
142	Developing models to strengthen the community role to solve the problems caused by COVID-19 in LMIC	0.73	0.66	0.58	0.66	0.73	0.67	0.66
143	Studying the social stigma related to COVID-19 and its effect on the outcomes in LMIC	0.88	0.50	0.57	0.78	0.80	0.71	0.70

144	Setting research priorities to improve sexual and psychological health of victims of domestic violence in LMIC countries during the COVID-19 pandemic	0.83	0.44	0.43	0.66	0.69	0.61	0.64
145	Studying how can we improve the coping mechanisms for those who lose their jobs during the COVID-19 pandemic in LMIC?	0.70	0.50	0.33	0.67	0.68	0.58	0.64
146	Studying how best to address the mistrust in the governments across LMIC during the pandemic situation such as COVID-19?	0.73	0.56	0.57	0.62	0.60	0.62	0.61
147	Systematic study to understand the sources and reasons for spreding COVID-19 misinformation in LMIC settings	0.75	0.56	0.52	0.67	0.58	0.61	0.61
148	Advancing the science around health communication strategies and community engagement in response and recovery efforts (how to build trust. address rumors. myths. infodemics)	0.79	0.72	0.62	0.79	0.65	0.71	0.71
149	Studying the impact of COVID-19 on sexual health	0.83	0.40	0.42	0.60	0.51	0.55	0.60
150	Developing specific guidelines for the treatment of COVID-19 in different LMIC contexts	0.76	0.78	0.54	0.76	0.74	0.72	0.70
151	Mobilizing research capacity across LMIC and "South-South" collaborations to search for an effective COVID-19 treatment	0.71	0.80	0.72	0.73	0.965	0.78	0.73
152	Characterizing clinical features of COVID-19 and their relationships with comorbidities and organ complications in LMIC settings	0.91	0.71	0.62	0.73	0.59	0.71	0.68
153	Studying the effects of COVID-19 on the recurrence rate of previous comorbidities in LMIC populations	0.82	0.61	0.54	0.68	0.55	0.64	0.62
154	Studying the inter-relation between existing comorbidities found mainly in LMIC (HIV. sickle cell disease. undernutrition. etc.). the occurrence of COVID-19 and post-COVID-19 condition	0.90	0.74	0.65	0.80	0.67	0.75	0.74
155	Characterizing the behaviours of LMIC populations after the effective measures against COVID-19 are relaxed to optimize recovery	0.81	0.56	0.44	0.56	0.45	0.57	0.59
156	Identifying resources and support necessary for effective home care of COVID-19 cases	0.85	0.71	0.49	0.74	0.66	0.69	0.68
157	Evaluating programmes that integrate HIV management into the context of COVID-19 in LMIC	0.84	0.58	0.48	0.64	0.70	0.65	0.65
158	Evaluating multidisciplinary approaches in the management of COVID-19 in LMIC health care settings	0.83	0.75	0.59	0.79	0.64	0.72	0.71
159	Developing large longitudinal cohorts to study long COVID-19 in LMIC settings	0.76	0.76	0.77	0.80	0.71	0.76	0.73
160	Systematic documentation and epidemiological assessment of the complications of COVID-19 infection in LMIC	0.93	0.74	0.68	0.77	0.68	0.76	0.74
161	Studying the effects of long-covid (post-covid syndrome) on mental health and wellbeing in different LMIC settings	0.93	0.71	0.66	0.78	0.79	0.77	0.76

162	Exploring strategies to mitigate the effects of long COVID-19 in LMIC populations	0.80	0.82	0.59	0.85	0.76	0.76	0.75
163	Exploring low-cost alternatives to mechanical ventilation in the treatment of severe clinical presentations of COVID-19 in LMIC?	0.83	0.82	0.58	0.82	0.81	0.77	0.76
164	Studying the effect of the COVID-19 pandemic on public health risks of other infectious diseases (e.g.. tuberculosis. malaria. AIDS) in LMIC settings?	0.92	0.65	0.51	0.77	0.66	0.70	0.69
165	Optimizing the management of drug-resistant tuberculosis in LMICs in the context of the COVID-19 pandemic	0.80	0.53	0.39	0.66	0.56	0.59	0.63
166	Evaluating the impact of the COVID-19 pandemic on the enrolment and treatment outcomes of drug-resistant tuberculosis cases in LMICs	0.88	0.54	0.39	0.63	0.51	0.59	0.63
167	Studying if there are unforeseen effects of the TB vaccines on COVID-19. and COVID-19 vaccines on TB	0.74	0.46	0.44	0.58	0.37	0.52	0.58
168	Evaluating the effects of the traditional Chinese medicine on treatment of COVID-19 in LMIC	0.55	0.45	0.43	0.57	0.43	0.49	0.55
169	Evaluating the effects of traditional medicine in LMIC contexts as a complementary therapy for COVID-19	0.66	0.49	0.54	0.65	0.45	0.56	0.56
170	Studying the effect of COVID-19 on childhood immunisations in LMIC	0.90	0.56	0.54	0.74	0.63	0.67	0.65
171	Developing scientific understanding whether it is rational to vaccinate children and young people in LMIC against COVID-19	0.76	0.61	0.58	0.63	0.61	0.64	0.63
172	Industrial research to enhance the vaccine manufacturing process in LMIC settings	0.73	0.75	0.54	0.72	0.75	0.70	0.68
173	Studying what are the barriers to access COVID-19 vaccination among the undocumented migrant workers in LMIC settings	0.73	0.70	0.44	0.70	0.82	0.68	0.68
174	Developing scientific understanding whether it is rational to provide booster doses of vascines against COVID-19 in LMIC	0.72	0.64	0.56	0.67	0.54	0.63	0.61
175	Studying the impact of vaccination of children and young people on school outbreaks	0.91	0.73	0.56	0.78	0.64	0.72	0.71
176	Document and analyse the anti-SARS-CoV-2 antibody response to vaccination in LMIC	0.82	0.69	0.62	0.67	0.56	0.67	0.67
177	Document and analyse COVID-19 vaccine side effects in LMIC populations	0.90	0.65	0.61	0.70	0.53	0.68	0.66
178	Studying how long does protective immunity last in vaccinated people in LMIC populations	0.87	0.81	0.73	0.79	0.62	0.76	0.75
179	Studying the effectiveness of different vaccines in LMIC and explore whether there are any differences vs. HIC	0.90	0.72	0.64	0.81	0.64	0.74	0.73
180	Studying factors that determine vaccine hesitancy in LMIC settings	1.00	0.83	0.66	0.92	0.80	0.84	0.83

181	Evaluating the positive effect of a peer-model of vaccine promotion and education. delivered through an Extension system. on vaccine uptake?	0.79	0.69	0.50	0.76	0.66	0.68	0.66
182	Evaluating the impact of epidemiological measures in different LMIC settings on vaccine uptake and COVID-19 outcomes	0.77	0.62	0.58	0.69	0.54	0.64	0.60
183	Development and evaluation of effective interventions to decrease vaccine hesitancy in general population	0.95	0.90	0.70	0.88	0.77	0.84	0.83
184	Development and evaluation of effective interventions to improve health care provider's COVID-19 knowledge and communication skills	0.98	0.69	0.48	0.84	0.59	0.71	0.72
185	Addressing the effect of the anti-vaccine misinformation campaigns on the prolongation of anti-COVID-19 measures	0.67	0.58	0.46	0.60	0.48	0.56	0.56
186	Quantifying the effects of conspiracy theories on vaccine acceptance and hesitancy in LMIC	0.65	0.66	0.57	0.56	0.49	0.59	0.58
187	Identifying factors that drive COVID-19 vaccine acceptance in LMIC population	0.97	0.87	0.65	0.84	0.64	0.79	0.78
188	Setting research priorities on expanded program immunization in LMIC during the COVID-19 pandemic to prevent reversals in coverage for other infectious diseases.	0.84	0.61	0.46	0.69	0.63	0.65	0.63
189	Assessing the influence of cultural and spiritual beliefs on vaccine hesitancy in LMIC	0.88	0.66	0.55	0.69	0.66	0.69	0.68
190	Comparing the level of public knowledge on COVID-19 pandemic and the success of vaccination programs among different populations in LMIc	0.91	0.74	0.48	0.73	0.74	0.72	0.72
191	A multinational study to understand social factors that could have led families to miss immunizations	0.80	0.62	0.44	0.68	0.69	0.65	0.63
192	Identifying factors that influence decision-making at the population level in LMIC for adhering with public health advice	0.91	0.81	0.65	0.80	0.77	0.79	0.78

Supplementary Table 2. Information on the study authors, who include 52 scorers and 17 management committee group (MCG) members (two MCG members also provided the scores).

First name	Last name	Sex	Country*	Region	Income	Role	Years of experience in global health	Job description	Submitted ideas	Received scoresheet	Provided scores
Ozren	Polašek	M	HR	Europe & Central Asia	High income	MCG member	17	Professor of Public Health	Y	Y	
Davies	Adeloye	M	UK	Europe & Central Asia	High income	MCG member	17	Research fellow in global health	Y	Y	
Peige	Song	F	CN	East Asia & Pacific	Upper middle income	MCG member	11	Professor of Global Health	Y	Y	Y
Kerri	Wazny	F	UK	Europe & Central Asia	High income	MCG member	12	Lead at Global Health Organization	Y	Y	
Kit	Chan	F	UK	Europe & Central Asia	High income	MCG member	20	Reader in Global Health	Y	Y	Y
Danladi	Adamu Bojude	M	NG	Sub-Saharan Africa	Lower middle income	Author	15	Associate Professor	Y	Y	Y
Sajjad	Ali	M	PK	South Asia	Lower middle income	Author	2	Researcher in Public Health	Y	Y	Y
Sheri	Bastien	F	NO	Europe & Central Asia	High income	Author	15	Professor of Public Health	Y	Y	Y
Francisco	Becerra	M	US	North America	High income	Author	40	Not disclosed	Y	Y	Y
Florencia	Borrescio-Higa	F	CL	Latin America & Caribbean	High income	Author	11	Associate Professor	Y	Y	Y
Sohaila	Cheema	F	QA	Middle East & North Africa	High income	Author	15	Assistant Dean for the Institute for Population Health	Y	Y	Y
Darien Alfa	Cipta	M	ID	East Asia & Pacific	Lower middle income	Author	5	Researcher in global mental health	Y	Y	Y
Smiljana	Cvjetković	F	RS	Europe & Central Asia	Upper middle income	Author	5	Lecturer in Public Health	Y	Y	Y
Lina	Díaz-Castro	F	MX	Latin America & Caribbean	Upper middle income	Author	6	Researcher in Medical Sciences	Y	Y	Y
Bassey	Ebenso	M	UK	Europe & Central Asia	High income	Author	30	Lecturer in global health	Y	Y	Y

Omolade	Femi-Ajao	F	UK	Europe & Central Asia	High income	Author	7	Lecturer in global health	Y	Y	Y
Balasankar	Ganesan	M	HK/CN	East Asia & Pacific	High income	Author	3	Researcher in global health	Y	Y	Y
Anton	Glasnović	M	HR	Europe & Central Asia	High income	Author	2	Assistant Professor	Y	Y	Y
Longtao	He	M	CN	East Asia & Pacific	Upper middle income	Author	8	Associate professor in health sociology	Y	Y	Y
Jean-Michel	Heraud	M	SN	Sub-Saharan Africa	Lower middle income	Author	15	Virologist	Y	Y	Y
Chinonso	Igwesi-Chidobe	F	NG	Sub-Saharan Africa	Lower middle income	Author	18	Senior Lecturer in Community Physiotherapy/Rehabilitation and Global Health	Y	Y	Y
Per Ole	Iversen	M	NO	Europe & Central Asia	High income	Author	15	Professor in clinical nutrition	Y	Y	Y
Bismeen	Jadoon	F	UK	Europe & Central Asia	High income	Author	6	Obstetrician and gynaecologist	Y	Y	Y
Abdulkarim	Jafar Karim	M	IQ	Middle East & North Africa	Upper middle income	Author	39	Assistant Professor	Y	Y	Y
Johra	Khan	F	SA	Middle East & North Africa	High income	Author	10	Assistant Professor	Y	Y	Y
Raaj Kishore	Biswas	M	AU	East Asia & Pacific	High income	Author	4	Biostatistician	Y	Y	Y
Giuseppe	Lanza	M	IT	Europe & Central Asia	High income	Author	2	Researcher in global mental health	Y	Y	Y
Shaun Wen Huey	Lee	M	MY	East Asia & Pacific	Upper middle income	Author	12	Associate Professor	Y	Y	Y
You	Lee	F	CN	East Asia & Pacific	Upper middle income	Author	5	Researcher in infectious diseases epidemiology	Y	Y	Y
Li-Lin	Liang	F	TW	East Asia & Pacific	High income	Author	2	Associate Professor of Health Economics	Y	Y	Y
Mat	Lowe	M	GM	Sub-Saharan Africa	Low income	Author	7	Director. Society for the Study of Women's Health	Y	Y	Y
Mohammad Mainul	Islam	M	BD	South Asia	Lower middle income	Author	20	Professor. Department of Population Sciences	Y	Y	Y
Ana	Marušić	F	HR	Europe & Central Asia	High income	Author	15	Professor of Anatomy and Research Methodology	Y	Y	Y
Suleiman	Mshelia	M	NG	Sub-Saharan Africa	Lower middle income	Author	7	Public health physician/	Y	Y	Y

								researcher			
Anthony Muchai	Manyara	M	KE	Sub-Saharan Africa	Lower middle income	Author	7	Research assistant/ project manager	Y	Y	Y
Mila Nu Nu	Htay	F	MY	East Asia & Pacific	Upper middle income	Author	6	Assistant Professor	Y	Y	Y
Michelle	Parisi	F	US	North America	High income	Author	1	Assistant professor	Y	Y	Y
Prince	Peprah	M	AU	East Asia & Pacific	High income	Author	Not disclosed	Not disclosed	Y	Y	Y
Emma	Sacks	F	US	North America	High income	Author	15	Associate Faculty. researcher	Y	Y	Y
Kabiru Olusegun	Akinyemi	M	NG	Sub-Saharan Africa	Lower middle income	Author	6	Professor of Microbiology & Infectious Diseases	Y	Y	Y
Fariba	Shahraki-Sanavi	F	IR	Middle East & North Africa	Lower middle income	Author	Not disclosed	Not disclosed	Y	Y	Y
Konstantin	Sharov	M	RU	Europe & Central Asia	Upper middle income	Author	12	Researcher in global health	Y	Y	Y
Elena S.	Rotarou	F	CL	Latin America & Caribbean	High income	Author	7	Assistant Professor	Y	Y	Y
Srđan	Stankov	M	RS	Europe & Central Asia	Upper middle income	Author	34	Chief of Department of microbiology	Y	Y	Y
Supriyatiningsih	Wenang	F	ID	East Asia & Pacific	Lower middle income	Author	8	Expert in Maternal and child health	Y	Y	Y
Benjamin	Chan	M	HK	East Asia & Pacific	High income	Author	3	University Dean and health educator	Y	Y	Y
Mark	Tremblay	M	CA	North America	High income	Author	26	Senior Scientist and Professor of Paediatrics	Y	Y	Y
Dialechti	Tsimpida	F	GB	Europe & Central Asia	High income	Author	8	Postdoctoral researcher	Y	Y	Y
Sandro	Vento	M	KH	East Asia & Pacific	Lower middle income	Author	13	Dean of Medical School	Y	Y	Y
Josipa	Vlasac Glasnovic	F	HR	Europe & Central Asia	High income	Author	2	Consultant in Haematology	Y	Y	Y
Liang	Wang	M	CN	East Asia & Pacific	Upper middle income	Author	3	Professor in Microbiology and Bioinformatics	Y	Y	Y
Xin	Wang	F	CN	East Asia & Pacific	Upper middle income	Author	5	Researcher in infectious diseases epidemiology	Y	Y	Y
Zhi Xiang	Ng	M	MY	East Asia & Pacific	Upper middle income	Author	11	Assistant Professor in Nutrition and Health	Y	Y	Y
Jianrong	Zhang	M	AU	East Asia & Pacific	High income	Author	9	Public health researcher	Y	Y	Y

Yanfeng	Zhang	M	CN	East Asia & Pacific	Upper middle income	Author	Not disclosed	Not disclosed	Y	Y	Y
Harry	Campbell	M	UK	Europe & Central Asia	High income	MCG member	38	University Professor in Epidemiology			
Mickey	Chopra	M	US	North America	High income	MCG member	28	Global Health Expert and Policy Maker at International Organizations			
Simon	Cousens	M	UK	Europe & Central Asia	High income	MCG member	27	University Professor in Medical Statistics			
Goran	Krstić	M	HR	Europe & Central Asia	High income	MCG member	2	Secretary General at ISoGH society			
Calum	Macdonald	M	UK	Europe & Central Asia	High income	MCG member	1	CHNRI software developer			
Parisa	Mansoori	F	UK	Europe & Central Asia	High income	MCG member	9	Global Health Expert at NIHR UK			
Smruti	Patel	F	US	North America	High income	MCG member	27	Independent Consultant in Global Health and Journal Editor			
Aziz	Sheikh	M	UK	Europe & Central Asia	High income	MCG member	25	Director of the Institute			
Mark	Tomlinson	M	ZA	Sub-Saharan Africa	Upper middle income	MCG member	22	University Professor in Global Health			
Alex	Tsai	M	US	North America	High income	MCG member	19	University Professor and journal editor			
Sachiyo	Yoshida	F	CH	Europe & Central Asia	High income	MCG member	14	Global Health Expert and Policy Maker at International Organizations			
Igor	Rudan	M	UK	Europe & Central Asia	High income	MCG member	25	University Professor in International Health			

*Abbreviations for countries and territories: HR-Croatia; UK-United Kingdom; CN-China; NG-Nigeria; PK-Pakistan; NO-Norway; US-United States of America; CL-Colombia; QA-Qatar; ID-Indonesia; RS-Serbia; MX-Mexico; HK-Hong Kong; IQ-Iraq; SA-Saudi Arabia; AU-Australia; SN-Senegal; IT-Italy; MY-Malaysia; TW-Taiwan; GM-The Gambia; BD-Bangladesh; KE-Kenya; RU-Russia; CA-Canada; KH-Cambodia; ZA-South Africa; CH-Switzerland.
